# Local Control after Locally Ablative, Image-Guided Radiotherapy of Oligometastases Identified by Gallium-68-PSMA-Positron Emission Tomography in Castration-Sensitive Prostate Cancer Patients (OLI-P)

**DOI:** 10.3390/cancers14092073

**Published:** 2022-04-21

**Authors:** Tobias Hölscher, Michael Baumann, Jörg Kotzerke, Klaus Zöphel, Frank Paulsen, Arndt-Christian Müller, Daniel Zips, Christian Thomas, Manfred Wirth, Esther G. C. Troost, Mechthild Krause, Steffen Löck, Fabian Lohaus

**Affiliations:** 1 Department of Radiotherapy and Radiation Oncology, Faculty of Medicine and University Hospital Carl Gustav Carus, Technische Universität Dresden, 01304 Dresden, Germany; esther.troost@uniklinikum-dresden.de (E.G.C.T.); mechthild.krause@uniklinikum-dresden.de (M.K.); steffen.loeck@oncoray.de (S.L.); fabian.lohaus@uniklinikum-dresden.de (F.L.); 2 National Center for Tumor Diseases (NCT), Partner Site Dresden, 01307 Dresden, Germany; joerg.kotzerke@uniklinikum-dresden.de (J.K.); christian.thomas@uniklinikum-dresden.de (C.T.); 3 OncoRay—National Center for Radiation Research in Oncology, Faculty of Medicine and University Hospital Carl Gustav Carus, Technische Universität Dresden, Helmholtz-Zentrum Dresden-Rossendorf, 01307 Dresden, Germany; michael.baumann@dkfz-heidelberg.de; 4 German Cancer Research Center (DKFZ), 69120 Heidelberg, Germany; 5 Department of Nuclear Medicine, Faculty of Medicine and University Hospital Carl Gustav Carus, Technische Universität Dresden, 01304 Dresden, Germany; klaus.zoephel@skc.de; 6 Department of Nuclear Medicine, Klinikum Chemnitz gGmbH, Medizincampus Chemnitz der TU Dresden, 09116 Chemnitz, Germany; 7 Department of Radiation Oncology, Medical Faculty and University Hospital Tübingen, 72001 Tübingen, Germany; frank.paulsen@uni-tuebingen.de (F.P.); arndt-christian.mueller@rkh-kliniken.de (A.-C.M.); daniel.zips@med.uni-tuebingen.de (D.Z.); 8 Department of Radiation Oncology, RKH-Kliniken Ludwigsburg, Academic Hospital of University Heidelberg, 69120 Ludwigsburg, Germany; 9 German Cancer Consortium (DKTK), Partner Site Tübingen, German Cancer Research Center (DKFZ), 69120 Heidelberg, Germany; 10 Department of Urology, Faculty of Medicine and University Hospital Carl Gustav Carus, Technische Universität Dresden, 01304 Dresden, Germany; manfred.wirth@uniklinikum-dresden.de; 11 German Cancer Consortium (DKTK), Partner Site Dresden, German Cancer Research Center (DKFZ), 69120 Heidelberg, Germany; 12 Helmholtz-Zentrum Dresden-Rossendorf, Institute of Radiooncology-OncoRay, 01304 Dresden, Germany

**Keywords:** prostatic neoplasms, prospective studies, radiotherapy, image-guided, radiosurgery, positron emission tomography, prostate-specific antigen, neoplasm metastasis, local control

## Abstract

**Simple Summary:**

In this clinical trial, 63 patients with a total of 89 prostate cancer metastases identified on PSMA-PET were included, none of them undergoing androgen deprivation therapy. We showed that local ablative radiotherapy controls >90% of the metastases, but progression at other sites is common after two years. Local ablative radiotherapy may be an option to at least temporarily avoid systemic therapy in selected patients.

**Abstract:**

Progression of prostate-specific antigen (PSA) values after curative treatment of prostate cancer patients is common. Prostate-specific membrane antigen (PSMA-) PET imaging can identify patients with metachronous oligometastatic disease even at low PSA levels. Metastases-directed local ablative radiotherapy (aRT) has been shown to be a safe treatment option. In this prospective clinical trial, we evaluated local control and the pattern of tumor progression. Between 2014 and 2018, 63 patients received aRT of 89 metastases (MET) (68 lymph node (LN-)MET and 21 bony (OSS-)MET) with one of two radiation treatment schedules: 50 Gy in 2 Gy fractions in 34 MET or 30 Gy in 10 Gy fractions in 55 MET. The mean gross tumor volume and planning target volume were 2.2 and 14.9 mL, respectively. The median follow-up time was 40.7 months. Local progression occurred in seven MET, resulting in a local control rate of 93.5% after three years. Neither treatment schedule, target volume, nor type of lesion was associated with local progression. Regional progression in the proximity to the LN-MET was observed in 19 of 47 patients with at least one LN-MET (actuarial 59.3% free of regional progression after 3 years). In 33 patients (52%), a distant progression was reported. The median time to first tumor-related clinical event was 16.6 months, and 22.2% of patients had no tumor-related clinical event after three years. A total of 14 patients (22%) had another aRT. In conclusion, local ablative radiotherapy in patients with PSMA-PET staged oligometastatic prostate cancer may achieve local control, but regional or distant progression is common. Further studies are warranted, e.g., to define the optimal target volume coverage in LN-MET and OSS-MET.

## 1. Introduction

The mainstay of treatment for locally confined prostate cancer (PCa) is radical prostatectomy or definitive radiation therapy [1]. Progression of prostate-specific antigen (PSA) levels after curative primary therapy is common in patients with prostate cancer. One in three patients having risk factors for progression after radical prostatectomy for prostate cancer will experience a PSA recurrence within 5 years [2]. After salvage radiotherapy, approximately 40% will develop further rising PSA levels within 6 years [3]. These patients have a high probability of having occult metastatic disease.

Modern molecular-based imaging methods, such as positron emission tomography using Gallium-68-labeled prostate-specific membrane antigen (PSMA) in combination with anatomical imaging, e.g., computed tomography (PSMA-PET-CT), may detect patients with metachronous oligometastatic disease at low PSA levels [4,5,6].

Several clinical trials showed that metastases-directed local ablative therapy is feasible and, in general, a safe option in this situation [7,8,9,10,11,12]. Local ablative radiotherapy (aRT) seems to bear a smaller risk of complications compared to metastases-directed surgery [8,13].

However, not all patients benefit from aRT. Despite the high sensitivity of PSMA-PET-CT for detecting oligometastatic disease in progressing PCa, there are several reports on patients who progress early after aRT to the PET-positive lesions with no or only minimal PSA response [10,11,13]. At least two mechanisms may lead to early progression: PET imaging may have been not sensitive enough to detect subclinical distant metastases, or circulating tumor cells already exist at the time of aRT. In addition, local failures in the primary tumor region might have been overlooked, for example, because the Gallium-68-PSMA tracer in the bladder masked tumor tissue next to the urethral anastomosis. This needs to be differentiated from the progression of the irradiated metastases because of biologically mediated radioresistance, underdosage, or incomplete coverage of the “true” target volume. At present, only little is known about the effect of target volume concepts, radiation dose, or additional systemic therapies in the treatment of oligometastatic prostate cancer [14,15].

Thus, we conducted the Gallium-68-PSMA-PET-CT-based, prospective OLI-P clinical trial exploring local ablative radiotherapy of up to five metachronous metastases in castration-sensitive PCa patients after curative primary therapy. The primary endpoint, reported earlier, was toxicity within two years after aRT [10]. Here we present the results on the predefined secondary endpoint, local-recurrence-free time of the irradiated metastases, and the time to the first tumor-related clinical event.

## 2. Materials and Methods

### 2.1. Trial Design

At two German centers, the non-randomized, phase-2 clinical trial “Effectiveness and Toxicity of Percutaneous High-dose Radiotherapy in Patients with **OLI**gometastases of **P**rostate Carcinoma” (OLI-P) recruited patients with PSA progression after local curative treatment (radical prostatectomy and/or radiation therapy), who had up to five PSMA-PET-avid metastases on Gallium-68-PSMA-PET-hybrid imaging. No histological confirmation of the metastases was obtained. Two experienced nuclear medicine physicians (K. Z. and J. K.) defined the metastases according to standard criteria [16,17]. All cases were discussed and confirmed in a multidisciplinary tumor board.

Following inclusion and exclusion criteria were required upon registration: up to five PSMA-PET-avid metastases of a previously curatively treated prostate cancer, no local tumor recurrence in the prostatic fossa or visceral metastases, no ongoing ADT, a PSA value below 10 ng/mL, and no severe comorbidity limiting life expectancy to less than five years. Concurrent treatment of the prostate bed was not performed. All participants provided written informed consent. The protocol was approved by the local Ethics Committees of both centers (EK 194052014). The trial was registered at clinicaltrials.gov (NCT02264379) and was conducted in accordance with the Declaration of Helsinki. A detailed description of the trial was published earlier [10].

### 2.2. Radiation Therapy

For delineation of target volumes, the diagnostic PET-CT imaging was fused with the planning CT. The metastatic lesion was identified in both modalities, and the segmentation of the gross tumor volume (GTV) was performed manually in the planning CT. A margin of 2 mm was added to create the clinical target volume (CTV) considering subclinical tumor spread and corrected for anatomical boundaries. An additional margin of 4 mm for SABR or 6 mm for 3D-CRT was applied to create the planning target volume (PTV).

**Figure 1:**
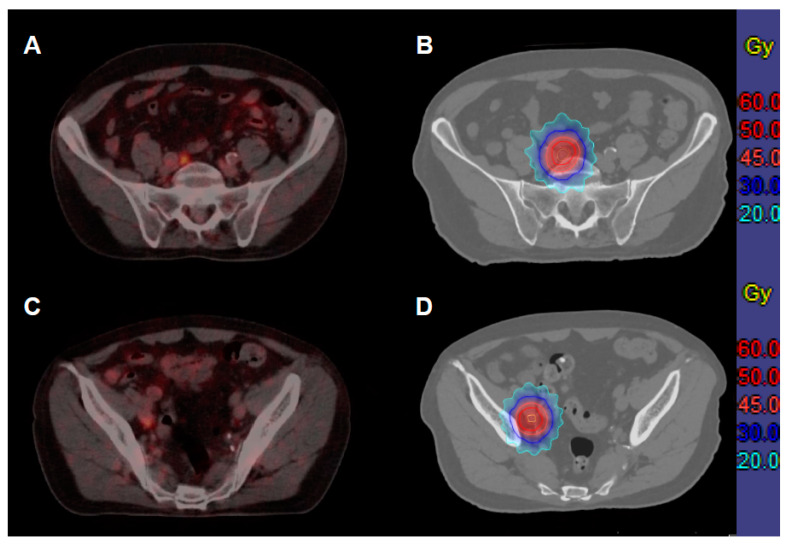
A 75-year-old patient with intermediate-risk prostate cancer (PSA 13.35 ng/mL, Gleason’s score 4 + 3 = 7) received primary radiation therapy (76 Gy in 2 Gy fractions). After three years, he developed a biochemical failure (3.4 ng/mL), and the PSMA-PET-CT showed two iliac lymph node metastases (**A**,**C**), which were treated with ablative radiotherapy (50 Gy in 2 Gy fractions, (**B**,**D**) gross tumor volume depicted in yellow, clinical target volume in orange, planning target volume in red. Until five years after treatment (end of study), no recurrent disease was detected.

A linear-accelerator-based, local ablative radiation therapy (aRT) to all PSMA-PET-avid metastases was performed. The study protocol predefined two different fractionation schedules that were feasible for most patients and left the decision regarding the treatment schedule to the treating radiation oncologist. Either stereotactic ablative radiotherapy (SABR) with a total dose of 30 Gy in three fractions of 10 Gy (80% isodose line encompassing the PTV) or conventionally fractionated, three-dimensional conformal radiotherapy (3D-CRT) with 50 Gy in 25 fractions of 2 Gy (according to ICRU 50/62) was applied (Figure 1). Smaller metastases were preferably treated with SABR. 3D-CRT was recommended for larger target volumes or next to critical organs at risk (e.g., bowel or spinal cord).

The dose recommendations for organs at risk published by the AAPM 101 task group were taken into account [18]. In case of an overlap of the PTV with the previously irradiated high dose, the increased risk of late toxicity was estimated, and the conventionally fractionated schedule was often recommended. Daily image-guided high-precision radiotherapy using an in-room CT scanner or cone-beam CT was performed.

### 2.3. Follow-Up

Follow-up visits were scheduled 3, 6, 12, 18, and 24 months after treatment and yearly thereafter. During follow-up, the clinical status, the use of androgen deprivation therapy, and the PSA values were assessed. All events and side-effects were prospectively scored at these time points according to the Common Terminology Criteria for Adverse Events scoring system (CTCAE V4.0). Radiological examinations were performed on request or at PSA progression only. Restaging with PSMA-PET-CT and a multidisciplinary tumor board discussion were recommended but not mandatory. In general, the referring physician started ADT in case of further PSA progression according to standard guidelines.

### 2.4. Statistics

The secondary endpoints considered in this manuscript were local progression-free time and time to the first tumor-related clinical event. Time to local progression was calculated as the time from the start of aRT to the first sign of radiological progression of the treated metastasis, irrespective of PSA progression or the start of ADT. Time to first tumor-related clinical event was defined as the time from the start of aRT to the first occurrence of systemic, regional, or local progression, or the start of ADT, irrespective of PSA progression. Regional progression was evaluated for LN-METs only. In case of detection of new LN-MET during follow-up, regional progression was considered if the LN-MET was in proximity of a previously irradiated lymph node metastasis.

Descriptive statistics were used to summarize patient and treatment-related data. The clinical endpoints were evaluated using the Kaplan–Meier method. Univariate Cox regression was performed to assess the influence of selected patient-, tumor-, and treatment-related factors. Factors with a *p*-value < 0.1 in univariate analyses were included in multivariate Cox regression analyses for time to the first tumor-related clinical event but not for local progression-free time since only seven events occurred. GTV and PTV were compared between patient subgroups using the Mann–Whitney-U test. Two-sided tests were performed, and *p*-values ≤ 0.05 were considered statistically significant. All analyses were performed with SPSS 27 software (IBM Corporation, Armonk, NY, USA). The data set was extracted from the clinical trial database for this analysis on 15 October 2021.

## 3. Results

Between 2014 and 2018, 72 patients were recruited. Nine patients were excluded from the analyses since they did not fulfill inclusion criteria (*n* = 5) or were considered to be at high risk of severe toxicity of aRT (*n* = 4), considering previous irradiation (e.g., proximity to small bowel or rectal wall with cumulative doses exceeding >100 Gy). In these cases, no aRT was performed after individual discussion with the patients.

The remaining 63 patients had 89 PSMA-PET-avid metastatic lesions (see Table 1). Most patients had one PSMA-PET-avid metastasis (*n* = 45, 71.4%), just seven participants (11.1%) had 3 or 4 metastases. The majority of patients had initially been treated with radical prostatectomy (*n* = 60; 95.2%), and 44 patients (69.8%) had a history of pelvic radiation therapy.

A total of 68 lymph node (LN-MET) and 21 bony (OSS-MET) metastases were treated with aRT (Table 2). The mean GTV and PTV were 2.2 and 14.9 mL, respectively. The GTVs and PTVs of the OSS-MET were significantly larger than the volumes of the LN-MET (GTV: 2.9 vs. 0.9 cm^3^, *p* < 0.001; PTV: 16.8 vs. 8.9 cm^3^, *p* = 0.014).

A total of 34 and 55 MET were treated with 3D-CRT and SABR, respectively. 3D-CRT was predominantly used for pelvic lymph node metastases (25 of 34 lesions; 73.5%). The GTVs of the conventionally fractionated metastases were not statistically different from the SABR group. As a result of the larger CTV to PTV margin for 3D-CRT, the PTVs of SABR were smaller than those of 3D-CRT (GTV: *p* = 0.14, PTV: *p* = 0.025).

The median follow-up time was 40.7 months. PSA progression or the start of ADT occurred in 47 patients after a median interval of 13.2 months (95% CI: 10.6–15.8), as reported earlier [10]. At this time point, 35 patients (74%) had PSMA-PET imaging for restaging.

**Figure 2:**
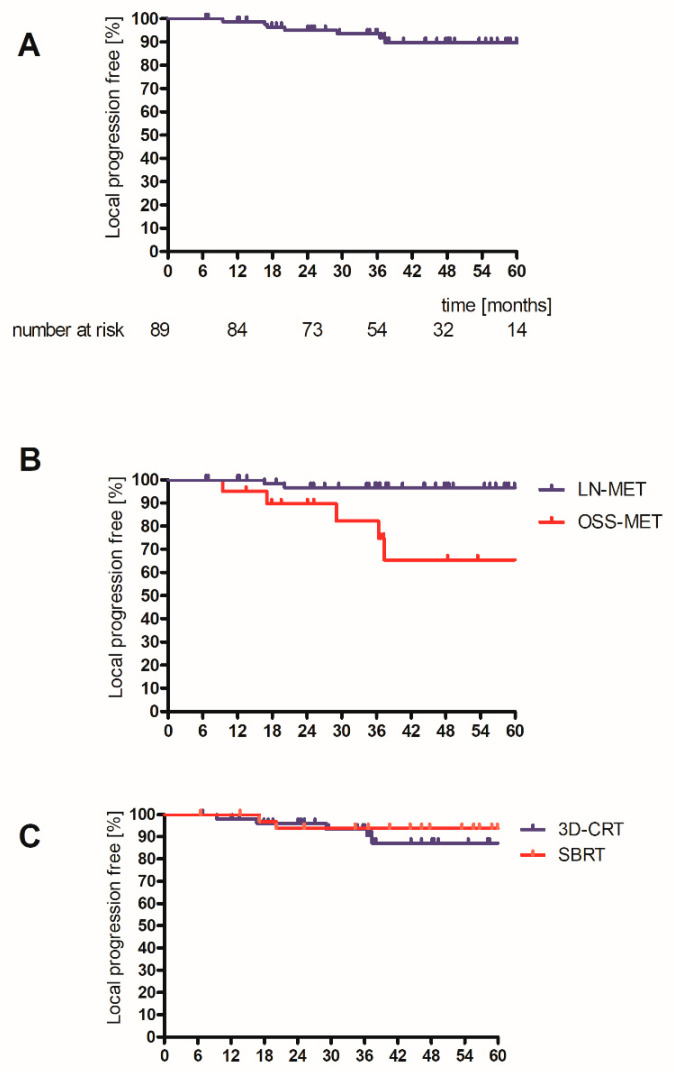
Time to local progression for entire cohort (**A**), for type of MET (**B**), and for SBRT or 3D-CRT (**C**). Log-rank: type of lesion: *p* < 0.001; treatment schedule *p* = 0.55. OSS-MET: osseous metastasis, LN-MET: lymph node metastasis, 3D-CRT: 3D-conformal radiotherapy, SBRT: stereotactic body irradiation.

After three years, 93.5% of metastases were free of local progression (Figure 2A). Five OSS-METs progressed, as compared to two LN-METs (Figure 2B, log-rank test *p* < 0.001). Four locally recurrent lesions were in non-spinal bony metastases. These were larger than other metastases (patients 4, 6, and 7, see Table 3). The selected treatment schedule (SBRT and 3D-CRT) did not statistically significantly influence the risk of local progression (Figure 2C, log-rank test *p* = 0.55), while GTV and PTV showed a statistical trend (log-rank: GTV > median: *p* = 0.079; PTV > median *p* = 0.060).

**Figure 3:**
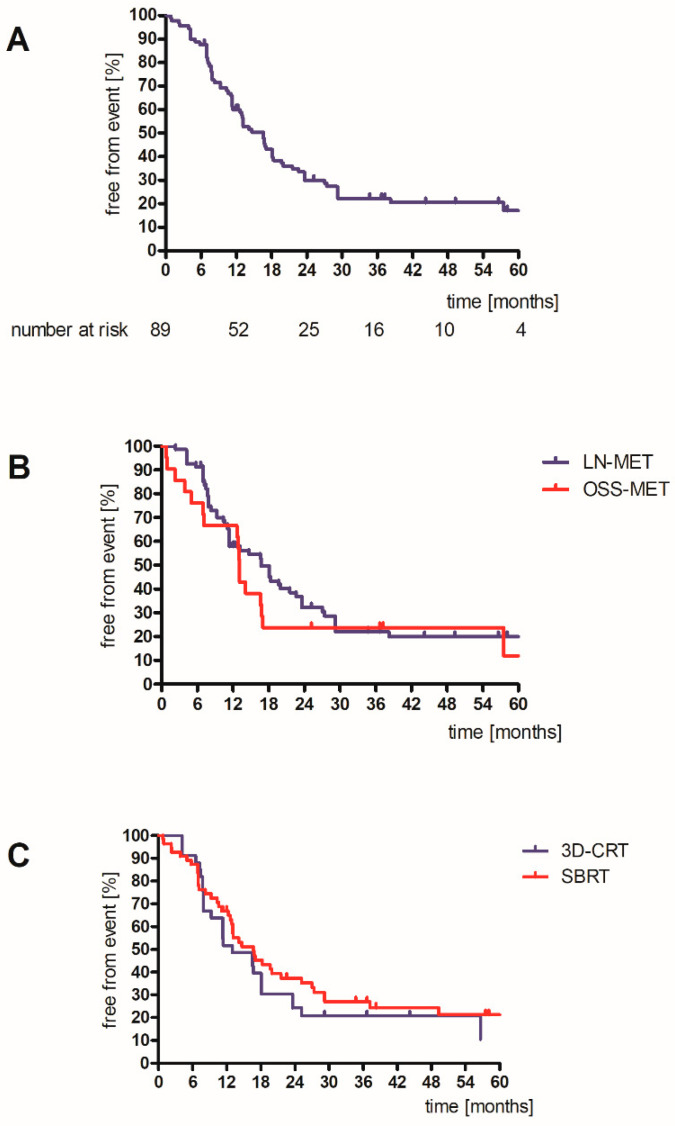
Time to first tumor-related clinical event for entire cohort (**A**), for type of MET (**B**), and for SBRT or 3D-CRT (**C**). Log-rank: type of lesion: *p* = 0.36; treatment schedule *p* = 0.45. OSS-MET: osseous metastasis, LN-MET: lymph node metastasis, 3D-CRT: 3D-conformal radiotherapy, SBRT: stereotactic body irradiation.

Any tumor-related event was observed in 69 lesions, resulting in a median failure-free time of 16.6 months (95% CI: 13.2–19.9). After three years, 16 lesions (actuarial 22.2%) were tumor-related event-free (Figure 3A). No differences in event-free time were observed between OSS-MET vs. LN-MET and between the two radiation treatment schedules (SBRT and 3D-CRT) (Figure 3B,C). The GTV and PTV showed a statistical trend (log-rank: GTV > median: *p* = 0.057; PTV > median *p* = 0.060). Regional progression in the proximity to the LN-MET was observed in 19 of 47 patients with at least 1 LN-MET. No regional progression was observed in 59.3% of patients after three years.

**Figure 4:**
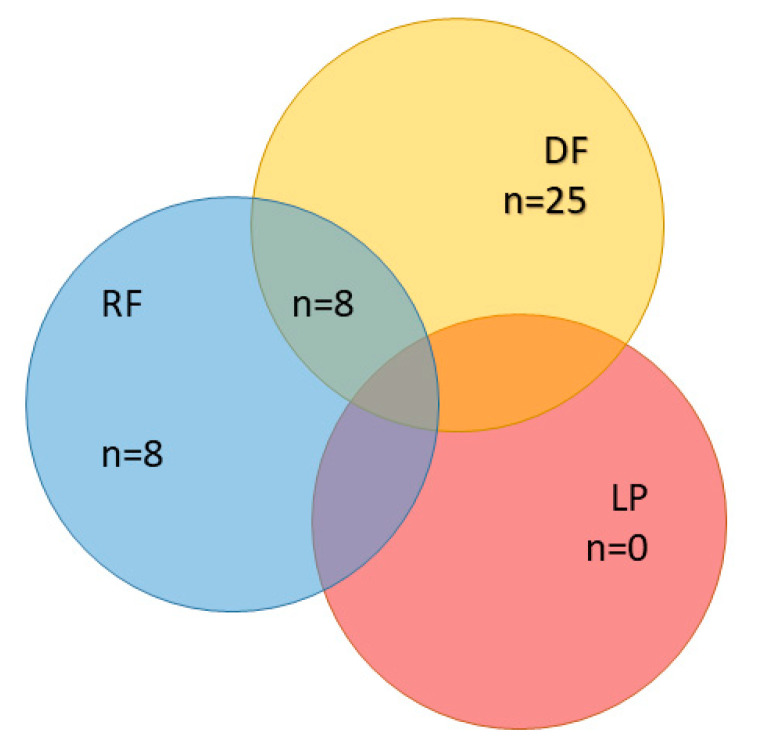
First tumor-related clinical event (per patient, *n* = 63), no event: *n* = 15, ADT as first tumor-related clinical event: *n* = 7 (not considered). ADT: androgen deprivation therapy, RF: regional failure; LP: local progression, DF: distant failure.

ADT was the first clinical event in 7 patients (11%), regional failure in 16 patients (25%), and distant failure in 33 patients (52%). In eight cases, regional and distant failure occurred simultaneously (Figure 4). Fourteen patients (22%) had another aRT in case of oligoprogressive disease.

In univariate analyses, time from PSMA-PET to aRT, type of lesion (LN-MET vs. OSS-MET), and GTV were statistically significantly correlated with the event of local progression (Table 4). As there were only seven events, no multivariate analysis for time to local progression was performed.

In univariate analysis time to first tumor-related event was significantly associated with initial tumor grading (ISUP grade, *p* = 0.010) and PSA response to aRT (*p* < 0.001), while time from initial treatment (*p* = 0.059), age at aRT (*p* = 0.069) and GTV (*p* = 0.052) showed a statistical trend. These factors were analyzed in a multivariate Cox regression analysis (Table 5). GTV and PSA response to aRT were statistically significant factors for the occurrence of a tumor-related event. Initial tumor grading (ISUP grade) missed the level of statistical significance (*p* = 0.065).

## 4. Discussion

Local ablative radiotherapy of PSMA-PET-identified metastases in patients with metachronous oligorecurrent hormone-sensitive prostate cancer is feasible and yields a high local control rate. In a relevant subset of patients, the onset of systemic therapy can be effectively delayed. Notwithstanding, progressive disease, mostly distantly progressing metastases, is common within months.

Local control in the current study was very high. There were just seven local recurrences of irradiated metastases, and none were the first clinical event. Local control was not different for the two radiotherapy schedules. This is interesting, as both RT regimens have different biological effectiveness, and 3D-CRT with 50 Gy is not generally considered a local ablative dose able to eradicate macroscopic disease.

Local control was excellent in all metastatic lesions except for non-spinal MET. Those bony metastases, primarily occurring in pelvic bones, were larger, and in this situation, safety margins of SABR or the applied dose were probably not sufficient. In our study, MR-imaging was not used to determine the local extent of non-spinal OSS-MET as recently proposed to assess the extent of the metastasis and to improve interobserver homogeneity of the GTV [19,20]. In consequence, our data support the now published consensus guideline for SBRT in non-spinal bony metastases that recommends sufficiently large GTV to CTV margins of up to 10 mm [21].

For SABR, the optimal dose concept has not yet been defined. A recent clinical trial showed high local control rates of three different dose concepts (including 3 × 10 Gy, as applied here) for SABR of non-spinal bone and lymph node metastases [22]. Another small randomized trial comparing local control after a single dose and a three-fraction SABR regime in bone metastases of various tumors entities showed superior local control of the single-dose SABR [23]. The authors did not report detailed information on the staging procedures and size of the treated metastases. Therefore, the observed differences might be explained by differences in the size of treated lesions. A recently published meta-analysis showed superior local control with increased toxicity for single-dose versus fractionated SABR or fractionated radiotherapy [15]. Again, the effect of confounding factors such as different primary tumors (other than prostate) or volume of metastases cannot be excluded. The small sample size and low number of events do not allow for statistically sound subgroup analyses, and firm conclusions on the role of the applied radiation treatment schemes cannot be made. Reasons for the similar response to the radiation schedules might be the limited time of follow-up or the overall low tumor burden in the trial’s population that can in fact be controlled by the applied doses. The target volumes (at a median PSA value of 2 ng/mL) are rather small, therefore local control can be achieved in these cases.

In the primary treatment of prostate cancer, dose escalation improves the PSA response rate and local control [24]. However, this advantage was detectable after a follow-up time of more than two years, only [25]. This indicates that local control can be achieved with standard doses for several years in the majority of patients without systemic therapy, even for small macroscopic tumors. In addition, for elective target volumes, treatment doses of 45–50 Gy may be sufficient to improve local control in the vast majority of patients [26]. The beneficial effect of irradiation of elective volume in primary high-risk PCa was, however, not detectable earlier than three years after treatment, possibly caused by adjuvant androgen deprivation therapy.

In our study, regional progression has been observed for one in three patients after aRT of lymph node metastases. We performed local ablative aRT of the involved lesion only, and locoregional control may potentially be improved by extending the target volume to elective areas [27]. A large retrospective study suggested that elective nodal radiotherapy (in combination with ADT, given in 60% of patients) reduces the risk of nodal recurrences compared to SBRT. However, the risk of severe adverse events was increased in patients having elective nodal irradiation of larger treatment volumes, and short treatment schedules such as SABR-techniques are not established for those larger treatment volumes [28]. Francolini et al. also showed promising local control rates after SABR of pelvic lymph node metastases of oligorecurrent prostate cancer patients [29]. At a median follow-up of 20 months after SBRT of up to three pelvic lymph nodes, 61% of the patients had radiological evidence of relapse, with a median disease-free survival (DFS) of 15 months. A recent large retrospective cohort of 394 patients with oligorecurrent prostate cancer was analyzed regarding the PSA recurrence-free survival of treating an elective target volume compared to metastasis-directed therapy only. In a subgroup of 278 patients having no local recurrence in the prostate bed, the addition of ADT, treatment of an elective volume, and other tumor-related factors were statistically significant in multivariate analyses [30]. Late toxicity was significantly increased due to the larger target volumes (grade 2 gastrointestinal or genitourinary toxicity: 1.9% in aRT vs. 19.2% in elective volume radiotherapy).

Lépinoy et al. retrospectively analyzed 62 patients with choline PET staged oligometastatic nodal recurrence after curative primary therapy of prostate cancer [31]. Either SABR or extended field radiotherapy was applied. Patients having elective treatment had a statistically significantly lower probability of treatment failure. The influence of ADT on the result remains unclear, as 48% of patients selected for extended field radiotherapy had ADT, compared to 24% in the SABR treatment group. Our results support the notion that different target volume concepts for oligorecurrent lymph node metastases in prostate cancer are worth to be evaluated prospectively [32].

Furthermore, the role of early ADT in metachronous oligorecurrent prostate cancer patients is unknown. A randomized study showed that local ablative therapy of oligometastatic disease may allow postponing the start of ADT in patients with hormone-sensitive prostate cancer [8]. Patients are aware of the various potential adverse effects of ADT and ask for strategies to at least temporarily avoid the decline in health-related quality-of-life effects of a long-term androgen deprivation therapy [33,34,35]. In the case of pelvic synchronous oligorecurrent prostate cancer, ADT may improve long-term outcomes. However, no randomized trials have been published yet [36]. Retrospective data suggested that ADT, in combination with local ablative therapy of oligometastases in prostate cancer, improved biochemical control significantly, but the long-term benefit is still unclear [30,37]. Several patients prefer to delay the onset of ADT to maintain their quality of life. The effect of metastases-directed therapy on clinically more relevant endpoints such as overall survival is unknown. Relevant clinical trials are ongoing to highlight the role of ADT in this situation, e.g., [38].

The prospective Oli-P phase-2 clinical trial was performed at two German centers. A stringent predefined patient selection, the use of state-of-the-art Gallium-68-PSMA-PET-CT imaging as an initial staging procedure, and the consistent use of two predefined treatment schedules are to be highlighted. According to protocol, no ADT was applied until progression. However, several limitations of the presented data are to be mentioned. As a secondary analysis of a prospective non-randomized trial, the results are hypotheses generating only. Radiological examinations (i.e., PSMA-PET-CT hybrid imaging) were not scheduled on a regular basis during follow-up but were performed in case of PSA progression only, albeit for a relevant subset of patients (74%). As there were only a few cases of local progression, and all occurred after clinical progression, no statistically sound conclusions can be drawn on the optimal dose volume concepts or selection of patients who benefit from aRT.

## 5. Conclusions

The OLI-P-clinical trial demonstrated that metastasis-directed radiotherapy without simultaneous androgen deprivation is a promising concept in selected patients with metachronous oligometastatic hormone-sensitive prostate cancer. Local ablative radiotherapy of PSMA-PET staged metastases demonstrated a high local control rate.

However, regional progression in adjacent LN-METs or metastatic progression was common. Further studies are warranted, e.g., to define optimal radiation dose and target volume concepts for an increase in local control, especially in non-spinal bone metastases, and to select patients who benefit from early systemic intervention.

## Figures and Tables

**Table 1:** Patient, tumor, and treatment characteristics (*n* = 63).

Variable		Number (%), Unless Specified
Age at aRT in years (median (range))		72 (52–84)
PSA at first diagnosis in ng/mL (median (range))		10.7 (1.1–158)
Initial NCCN risk (*n* = 1: missing)	Intermediate	19 (30%)
	High	9 (14%)
	Very high	26 (41%)
	Locoregional	8 (13%)
Initial ISUP score (*n* = 1: missing)	1–2	30 (48%)
	3	14 (22%)
	4	10 (16%)
	5	8 (13%)
Primary local treatment	Radical prostatectomy	60 (95%)
	Primary radiotherapy/ postop. radiotherapy	3 (5%)/ 43 (68%)
	Dose in Gy (median (range))	66.0 (64–76)
Time from initial treatment in months (median (range))		56 (4–201)
PSA at inclusion in ng/mL (median (range))		2.2 (0.2–8.9)
Time from PSMA-PET to aRT in days (median (range))		42 (14–192)
Number of treated metastases (per patient) (68 LN-MET, 21 OSS-MET)	*n* = 1	45 (71%)
	*n* = 2	11 (17%)
	*n* = 3	6 (10%)
	*n* = 4	1 (2%)
Type of metastases (per patient)	LN-MET	43 (68%)
	OSS-MET	16 (25%)
	Both	4 (6%)

aRT: ablative radiotherapy, PSA: prostate-specific antigen, NCCN: National Comprehensive Cancer Network, ISUP: International Society of Urological Pathology, PSMA: prostate-specific membrane antigen, PET: positron emission tomography, LN-MET: lymph node metastasis, OSS-MET: osseous metastasis.

**Table 2:** Baseline characteristics of metastatic lesions (*n* = 89).

Variable		3D-CRT (50 Gy)	SBRT (30 Gy)	All
Localization of lesions	Non-spinal OSS-MET: M1b	0	13 (14.6%)	13 (14.6%)
	Spinal OSS-MET: M1b	2 (2.2%)	6 (6.7%)	8 (9.0%)
	Pelvic LN-MET: N1	25 (28.1%)	31 (34.8%)	56 (62.9%)
	Paraaortal LN-MET: M1a	7 (7.9%)	5 (5.6%)	12 (14.6%)
Treatment schedule	3D-CRT/SBRT	34 (38%)	55 (62%)	
Volume of lesions	GTV (median (cm^3^), range)	0.75 (0.1–10.2)	1.1 (0.1–19.0)	0.92 (0.1–19.0)
	PTV (median (cm^3^), range)	14.0 (4.5–65.6)	9.4 (1.7–64.0)	10.2 (1.7–65.6)
Lesion in previously irradiated volume?	No	20	44	64 (71.9%)
	Marginal	10	11	21 (23.6%)
	High-dose volume	4	0	4 (4.5%)

3D-CRT: 3D-conformal radiotherapy, SBRT: stereotactic body irradiation, OSS-MET: osseous metastasis, LN-MET: lymph node metastasis, GTV: gross tumor volume; PTV: planning target volume.

**Table 3:** Description of the seven local progressing lesions.

No. of Lesion	Lesion	Radiotherapy Schedule	GTV (cm^3^)	PTV (cm^3^)
1	OSS-MET thoracic vertebrae (T10/T11)	3D-CRT	2.1	16.6
2	LN-MET obturatoric fossa left	3D-CRT	4.5	26.4
3	LN-MET para aortal	SBRT	0.9	8.2
4	OSS-MET os ilium right	SBRT	10.8	40.7
5	OSS-MET os pubis right	SBRT	6.7	24.6
6	OSS-MET 8. rib dorsal right	SBRT	11.7	52
7	OSS-MET os ilium right	SBRT	19	64

GTV: gross tumor volume; PTV: planning target volume, 3D-CRT: 3D-conformal radiotherapy, SBRT: stereotactic body irradiation, OSS-MET: osseous metastasis, LN-MET: lymph node metastasis.

**Table 4:** Univariate Cox regression analysis for local progression and time to first tumor-related clinical event after aRT.

Variable	Time to Local Progression (7 Events)		Time to First Tumor-Related Clinical Event (69 Events)	
	*p*-Value	HR (95% CI)	*p*-Value	HR (95% CI)
PSA at initial diagnosis (ng/mL)	0.54	0.98 (0.92–1.04)	0.97	1.00 (0.99–1.01)
Risk NCCN (Intermediate/high/very high/locoregional)	0.53		0.36	
ISUP grade (1/2/3/4/5)	1.00		**0.010**	
Time from initial treatment to aRT (months)	0.72	1.00 (0.98–1.01)	**0.059**	0.99 (0.99–1.00)
PSA at inclusion (ng/mL)	0.96	0.99 (0.71–1.39)	0.58	1.03 (0.93–1.15)
Age at aRT (years)	0.54	0.97 (0.87–1.07)	**0.069**	0.97 (0.94–1.00)
Time from PSMA-PET to aRT (days)	**0.034**	1.016 (1.001–1.031)	0.22	1.01 (1.00–1.01)
Number of treated metastases (per patient)	0.38	0.08 (0–21.4)	0.58	1.12 (0.74–1.7)
Type of lesion (LN-MET/OSS-MET)	**0.007**	0.104 (0.02–0.54)	0.36	0.77 (0.45–1.34)
Treatment schedule (SBRT/3D-CRT)	0.55	0.61 (0.11–2.94)	0.46	1.21 (0.74–1.97)
Ln(GTV / cm^3^)	**0.001**	3.63 (1.65–7.99)	**0.052**	1.23 (1.00–1.51)
PSA Nadir (%)	0.30	0.99 (0.97–1.01)	**<0.001**	1.01 (1.006–1.014)

aRT: ablative radiotherapy, PSA: prostate-specific antigen, NCCN: National Comprehensive Cancer Network, ISUP: International Society of Urological Pathology, PSMA: prostate-specific membrane antigen, PET: positron emission tomography, LN-MET: lymph node metastasis, OSS-MET: osseous metastasis, GTV: gross tumor volume; in bold: values considered relevant (i.e., *p* < 0.1).

**Table 5:** Multivariate Cox regression analysis for time to first failure after aRT.

Variables	*p*-Value	HR (95% CI)	
ISUP grade (1/2/3/4/5)	0.065		
Time from initial treatment to aRT (months)	0.72	1.00	(0.99–1.01)
Age at aRT (years)	0.14	0.97	(0.93–1.01)
Ln(GTV/cm^3^)	**0.002**	1.45	(1.142–1.849)
PSA Nadir (%)	**<0.001**	1.02	(1.012–1.025)

aRT: ablative radiotherapy, ISUP: International Society of Urological Pathology, GTV: gross tumor volume; PSA: prostate-specific antigen; in bold: values considered statistically significant (i.e., *p* < 0.05).

## Data Availability

The data presented in this study are available on request from the corresponding author. The data are not publicly available due to privacy issues.

## References

[1] Hamdy F.C., Donovan J.L., Lane J.A., Mason M., Metcalfe C., Holding P., Davis M., Peters T.J., Turner E.L., Martin R.M. (2016). 10-Year Outcomes after Monitoring, Surgery, or Radiotherapy for Localized Prostate Cancer. N. Engl. J. Med..

[2] Parker C.C., Clarke N.W., Cook A.D., Kynaston H.G., Petersen P.M., Catton C., Cross W., Logue J., Parulekar W., Payne H. (2020). Timing of radiotherapy after radical prostatectomy (RADICALS-RT): A randomised, controlled phase 3 trial. Lancet.

[3] Ghadjar P., Hayoz S., Bernhard J., Zwahlen D.R., Hölscher T., Gut P., Polat B., Hildebrandt G., Müller A.-C., Plasswilm L. (2021). Dose-intensified Versus Conventional-dose Salvage Radiotherapy for Biochemically Recurrent Prostate Cancer After Prostatectomy: The SAKK 09/10 Randomized Phase 3 Trial. Eur. Urol..

[4] Deijen C.L., Vrijenhoek G.L., Schaake E.E., Vogel W.V., Moonen L.M., Pos F.J., van der Poel H.G., Borst G.R. (2021). PSMA-11-PET/CT versus choline-PET/CT to guide stereotactic ablative radiotherapy for androgen deprivation therapy deferral in patients with oligometastatic prostate cancer. Clin. Transl. Radiat. Oncol..

[5] Lecouvet E.F., Oprea-Lager D.-E., Liu Y., Ost P., Bidaut L., Collette L., Deroose C., Goffin K., Herrmann K., Hoekstra O.S. (2018). Use of modern imaging methods to facilitate trials of metastasis-directed therapy for oligometastatic disease in prostate cancer: A consensus recommendation from the EORTC Imaging Group. Lancet Oncol..

[6] Perera M., Papa N., Roberts M., Williams M., Udovicich C., Vela I., Christidis D., Bolton D., Hofman M., Lawrentschuk N. (2019). Gallium-68 Prostate-specific Membrane Antigen Positron Emission Tomography in Advanced Prostate Cancer—Updated Diagnostic Utility, Sensitivity, Specificity, and Distribution of Prostate-specific Membrane Antigen-avid Lesions: A Systematic Review and Meta-analysis. Eur. Urol..

[7] Phillips R., Shi W.Y., Deek M., Radwan N., Lim S.J., Antonarakis E.S., Rowe S.P., Ross A.E., Gorin M.A., Deville C. (2020). Outcomes of Observation vs Stereotactic Ablative Radiation for Oligometastatic Prostate Cancer: The ORIOLE Phase 2 Randomized Clinical Trial. JAMA Oncol..

[8] Ost P., Reynders D., Decaestecker K., Fonteyne V., Lumen N., De Bruycker A., Lambert B., Delrue L., Bultijnck R., Claeys T. (2018). Surveillance or Metastasis-Directed Therapy for Oligometastatic Prostate Cancer Recurrence: A Prospective, Randomized, Multicenter Phase II Trial. J. Clin. Oncol..

[9] Palma D.A., Olson R., Harrow S., Gaede S., Louie A.V., Haasbeek C., Mulroy L., Lock M., Rodrigues G.B., Yaremko B.P. (2020). Stereotactic Ablative Radiotherapy for the Comprehensive Treatment of Oligometastatic Cancers: Long-Term Results of the SABR-COMET Phase II Randomized Trial. J. Clin. Oncol..

[10] Hölscher T., Baumann M., Kotzerke J., Zöphel K., Paulsen F., Müller A.-C., Zips D., Koi L., Thomas C., Löck S. (2022). Toxicity and Efficacy of Local Ablative, Image-guided Radiotherapy in Gallium-68 Prostate-specific Membrane Antigen Targeted Positron Emission Tomography–staged, Castration-sensitive Oligometastatic Prostate Cancer: The OLI-P Phase 2 Clinical Trial. Eur. Urol. Oncol..

[11] Kneebone A., Hruby G., Ainsworth H., Byrne K., Brown C., Guo L., Guminski A., Eade T. (2018). Stereotactic Body Radiotherapy for Oligometastatic Prostate Cancer Detected via Prostate-specific Membrane Antigen Positron Emission Tomography. Eur. Urol. Oncol..

[12] Deek M.P., Taparra K., Dao D., Chan L., Phillips R., Gao R.W., Kwon E.D., Deville C., Song D.Y., Greco S. (2020). Patterns of Recurrence and Modes of Progression After Metastasis-Directed Therapy in Oligometastatic Castration-Sensitive Prostate Cancer. Int. J. Radiat. Oncol..

[13] Glicksman R.M., Metser U., Vines D., Valliant J., Liu Z., Chung P.W., Bristow R.G., Finelli A., Hamilton R., Fleshner N.E. (2021). Curative-intent Metastasis-directed Therapies for Molecularly-defined Oligorecurrent Prostate Cancer: A Prospective Phase II Trial Testing the Oligometastasis Hypothesis. Eur. Urol..

[14] Erler D., Brotherston D., Sahgal A., Cheung P., Loblaw A., Chu W., Soliman H., Chung H., Kiss A., Chow E. (2018). Local control and fracture risk following stereotactic body radiation therapy for non-spine bone metastases. Radiother. Oncol..

[15] Singh R., Lehrer E.J., Dahshan B., Palmer J.D., Sahgal A., Gerszten P.C., Zaorsky N.G., Trifiletti D.M. (2020). Single fraction radiosurgery, fractionated radiosurgery, and conventional radiotherapy for spinal oligometastasis (SAFFRON): A systematic review and meta-analysis. Radiother. Oncol..

[16] Toriihara A., Nobashi T., Baratto L., Duan H., Moradi F., Park S., Hatami N., Aparici C.M., Davidzon G., Iagaru A. (2019). Comparison of 3 Interpretation Criteria for 68Ga-PSMA11 PET Based on Inter- and Intrareader Agreement. J. Nucl. Med..

[17] Fanti S., Minozzi S., Morigi J.J., Giesel F., Ceci F., Uprimny C., Hofman M., Eiber M., Schwarzenbock S., Castellucci P. (2017). Development of standardized image interpretation for 68Ga-PSMA PET/CT to detect prostate cancer recurrent lesions. Eur. J. Pediatr..

[18] Benedict S.H., Yenice K.M., Followill D., Galvin J.M., Hinson W., Kavanagh B., Keall P., Lovelock M., Meeks S., Papiez L. (2010). Stereotactic body radiation therapy: The report of AAPM Task Group101: Stereotactic body radiation therapy: The report of TG101. Med Phys..

[19] Ilamurugu A., Chandrasekaran A., Ayyalusamy A., Satpathy S.P., Reddy J., Arora S., Subramanian S., Velayudham R. (2021). Volumetric and dosimetric impact of MRI in delineation of gross tumor volume of non-spinal vertebral metastases treated with stereotactic ablative radiation therapy. Cancer Radiothérapie.

[20] Raman S., Chin L., Erler D., Atenafu E.G., Cheung P., Chu W., Chung H., Loblaw A., Poon I., Rubenstein J. (2018). Impact of Magnetic Resonance Imaging on Gross Tumor Volume Delineation in Non-spine Bony Metastasis Treated With Stereotactic Body Radiation Therapy. Int. J. Radiat. Oncol..

[21] Nguyen T.K., Chin L., Sahgal A., Dagan R., Eppinga W., Guckenberger M., Kim J.H., Lo S.S., Redmond K.J., Siva S. (2021). International Multi-institutional Patterns of Contouring Practice and Clinical Target Volume Recommendations for Stereotactic Body Radiation Therapy for Non-Spine Bone Metastases. Int. J. Radiat. Oncol..

[22] Mercier C., Claessens M., Buys M.A., Gryshkevych S., Billiet C., Joye I., Van Laere S., Vermeulen P., Meijnders P., Löfman F. (2020). Stereotactic Ablative Radiation Therapy to All Lesions in Patients With Oligometastatic Cancers: A Phase 1 Dose-Escalation Trial. Int. J. Radiat. Oncol..

[23] Zelefsky M.J., Yamada Y., Greco C., Lis E., Schöder H., Lobaugh S., Zhang Z., Braunstein S., Bilsky M.H., Powell S.N. (2021). Phase 3 Multi-Center, Prospective, Randomized Trial Comparing Single-Dose 24 Gy Radiation Therapy to a 3-Fraction SBRT Regimen in the Treatment of Oligometastatic Cancer. Int. J. Radiat. Oncol..

[24] Viani G.A., Stefano E.J., Afonso S.L. (2009). Higher-Than-Conventional Radiation Doses in Localized Prostate Cancer Treatment: A Meta-analysis of Randomized, Controlled Trials. Int. J. Radiat. Oncol..

[25] Dearnaley D.P., Jovic G., Syndikus I., Khoo V., Cowan R., Graham J., Aird E.G., Bottomley D., Huddart A.R., Jose C.C. (2014). Escalated-dose versus control-dose conformal radiotherapy for prostate cancer: Long-term results from the MRC RT01 randomised controlled trial. Lancet Oncol..

[26] Murthy V., Maitre P., Kannan S., Panigrahi G., Krishnatry R., Bakshi G., Prakash G., Pal M., Menon S., Phurailatpam R. (2021). Prostate-Only Versus Whole-Pelvic Radiation Therapy in High-Risk and Very High-Risk Prostate Cancer (POP-RT): Outcomes From Phase III Randomized Controlled Trial. J. Clin. Oncol..

[27] Pinkawa M., Aebersold D.M., Böhmer D., Flentje M., Ghadjar P., Schmidt-Hegemann N.-S., Höcht S., Hölscher T., Müller A.-C., Niehoff P. (2021). Radiotherapy in nodal oligorecurrent prostate cancer. Strahlenther. Onkol..

[28] De Bleser E., Jereczek-Fossa B.A., Pasquier D., Zilli T., Van As N., Siva S., Fodor A., Dirix P., Gomez-Iturriaga A., Trippa F. (2019). Metastasis-directed Therapy in Treating Nodal Oligorecurrent Prostate Cancer: A Multi-institutional Analysis Comparing the Outcome and Toxicity of Stereotactic Body Radiotherapy and Elective Nodal Radiotherapy. Eur. Urol..

[29] Francolini G., Bellini C., Di Cataldo V., Detti B., Bruni A., Alicino G., Triggiani L., La Mattina S., D’Angelillo R., Demofonti C. (2021). Pattern of Recurrence After Stereotactic Radiotherapy in Prostate Cancer Patients With Nodal Pelvic Relapse. A Multi-Institutional Retrospective Analysis. Clin. Oncol..

[30] Kirste S., Kroeze S.G.C., Henkenberens C., Schmidt-Hegemann N.-S., Vogel M.M.E., Becker J., Zamboglou C., Burger I., Derlin T., Bartenstein P. (2021). Combining 68Ga-PSMA-PET/CT-Directed and Elective Radiation Therapy Improves Outcome in Oligorecurrent Prostate Cancer: A Retrospective Multicenter Study. Front. Oncol..

[31] Lépinoy A., Silva Y.E., Martin E., Bertaut A., Quivrin M., Aubignac L., Cochet A., Créhange G. (2018). Salvage extended field or involved field nodal irradiation in 18F-fluorocholine PET/CT oligorecurrent nodal failures from prostate cancer. Eur. J. Pediatr..

[32] Achard V., Bottero M., Rouzaud M., Lancia A., Scorsetti M., Filippi A.R., Franzese C., Jereczek-Fossa B.A., Ingrosso G., Ost P. (2020). Radiotherapy treatment volumes for oligorecurrent nodal prostate cancer: A systematic review. Acta Oncol..

[33] Nguyen P.L., Alibhai S.M.H., Basaria S., D’Amico A.V., Kantoff P.W., Keating N.L., Penson D.F., Rosario D.J., Tombal B., Smith M.R. (2015). Adverse Effects of Androgen Deprivation Therapy and Strategies to Mitigate Them. Eur. Urol..

[34] Duchesne G.M., Woo H.H., King M., Bowe S.J., Stockler M.R., Ames A., D’Este C., Frydenberg M., Loblaw A., Malone S. (2017). Health-related quality of life for immediate versus delayed androgen-deprivation therapy in patients with asymptomatic, non-curable prostate cancer (TROG 03.06 and VCOG PR 01-03 [TOAD]): A randomised, multicentre, non-blinded, phase 3 trial. Lancet Oncol..

[35] Alibhai S.M., Breunis H., Timilshina N., Johnston C., Tomlinson G., Tannock I., Krahn M., Fleshner N.E., Warde P., Canning S.D. (2010). Impact of Androgen-Deprivation Therapy on Physical Function and Quality of Life in Men With Nonmetastatic Prostate Cancer. J. Clin. Oncol..

[36] Supiot S., Vaugier L., Pasquier D., Buthaud X., Magné N., Peiffert D., Sargos P., Crehange G., Pommier P., Loos G. (2021). OLIGOPELVIS GETUG P07, a Multicenter Phase II Trial of Combined High-dose Salvage Radiotherapy and Hormone Therapy in Oligorecurrent Pelvic Node Relapses in Prostate Cancer. Eur. Urol..

[37] Kroeze S.G., Henkenberens C., Schmidt-Hegemann N.S., Vogel M.M.E., Kirste S., Becker J., Burger I.A., Derlin T., Bartenstein P., Eiber M. (2019). Prostate-specific Membrane Antigen Positron Emission Tomography–detected Oligorecurrent Prostate Cancer Treated with Metastases-directed Radiotherapy: Role of Addition and Duration of Androgen Deprivation. Eur. Urol. Focus.

[38] De Bruycker A., Spiessens A., Dirix P., Koutsouvelis N., Semac I., Liefhooghe N., Gomez-Iturriaga A., Everaerts W., Otte F., Papachristofilou A. (2020). PEACE V—Salvage Treatment of OligoRecurrent nodal prostate cancer Metastases (STORM): A study protocol for a randomized controlled phase II trial. BMC Cancer.

